# Acquired Elliptocytosis as a Manifestation of Myelodysplastic Syndrome Associated with Deletion of Chromosome 20q

**DOI:** 10.1155/2018/6819172

**Published:** 2018-02-01

**Authors:** Sukesh Manthri, Naresh K. Vasireddy, Sindhura Bandaru, Swati Pathak

**Affiliations:** ^1^Southern Illinois University, Springfield, IL, USA; ^2^Dr. NTR University of Health Sciences, Vijayawada, India

## Abstract

Elliptocytosis is commonly seen as a hereditary condition. We present a case of myelodysplastic syndrome (MDS) del(q20) variant with concomitant acquired elliptocytosis. A 73-year-old male with a history of prostate cancer presented to the hospital for evaluation of bleeding gums. Initial evaluation showed Hgb of 9.3 gm/dl, hematocrit of 28%, platelet count of 36,000 K/cmm, and WBC of 1.8 K/cmm with an ANC of 0.8 K/cmm. A slightly elevated bilirubin of 1.2 mg/dl spurred a hemolytic workup. Peripheral smear showed frequent elliptocytes, teardrop cells, schistocytes, and occasional spherocytes. Bone marrow biopsy did not show significant fibrosis to explain the elliptocytosis. Cytogenetics showed 20q deletion, and later, he was started on therapy for intermediate risk MDS. Bone marrow biopsy after completion of 6 cycles showed complete cytogenetic remission with significant improvement in elliptocytosis. Elliptocytosis in the setting of MDS has rarely been reported, and association with 20q deletion is even rarer. Animal studies have shown that haploinsufficiency of *L3MBTL1* contributes to some (20q−) myeloproliferative neoplasms and myelodysplastic syndromes by affecting erythroid differentiation. Our case report raises interesting questions: Does MDS with rarely reported elliptocytosis indicate a disease process that is different from the usual 20q deletion? Is haploinsufficiency of *L3MBTL1* responsible for this manifestation?

## 1. Introduction

Myelodysplastic syndrome (MDS) comprises a heterogeneous group of malignant stem cell disorders characterized by ineffective blood cell production and an increased risk of transformation to acute leukemia. The disease entity often produces many misshapen red blood cells from the bone marrow characterized by increased number of blasts, dysplastic cells, and ring sideroblast. Of these, elliptocytosis has been described previously in the literature but has not been described in conjunction with the many variants of MDS. Elliptocytosis is commonly seen as a hereditary condition. We present to you a case of MDS del(q20) variant with concomitant elliptocytosis (Table 1 shows patient's characteristics).

## 2. Case Report

**Figure 1:**
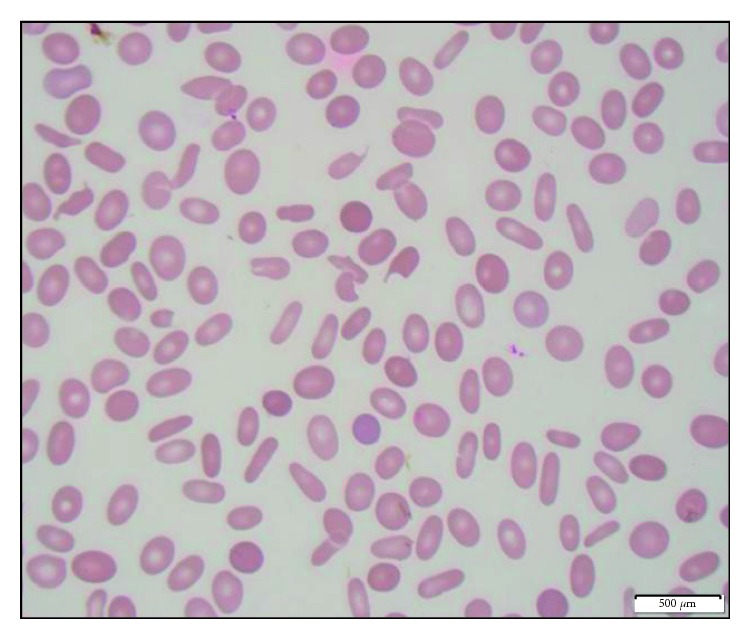
Peripheral smear showing frequent elliptocytes, tear drop cells, schistocytes, and occasional spherocytes.

A 73-year-old male with a history of prostate cancer (Gleason stage unknown) diagnosed in 2006 who underwent Tomo radiation to his prostate and pelvic area for 38 sessions presented to the hospital for evaluation of bleeding gums. He noticed a clot on his upper gum several weeks prior to presentation, which got worse and prompted him to visit his dentist. His dentist advised that it was not because of his dentition and asked him to go to emergency room for further evaluation. Initial evaluation showed Hgb of 9.3 gm/dl, hematocrit of 28%, platelet count of 36,000 K/cmm, and WBC of 1.8 K/cmm with an ANC of 0.8 K/cmm. A slightly elevated bilirubin of 1.2 mg/dl spurred a hemolytic workup. Reticulocyte % was 6.1%, LDH was 196 IU/L, and direct Coombs test was negative. Hematology was consulted for his pancytopenia. Subsequently, he was admitted for further workup. Peripheral smear in Figure 1 shows frequent elliptocytes, teardrop cells, schistocytes, and occasional spherocytes.

**Figure 2:**
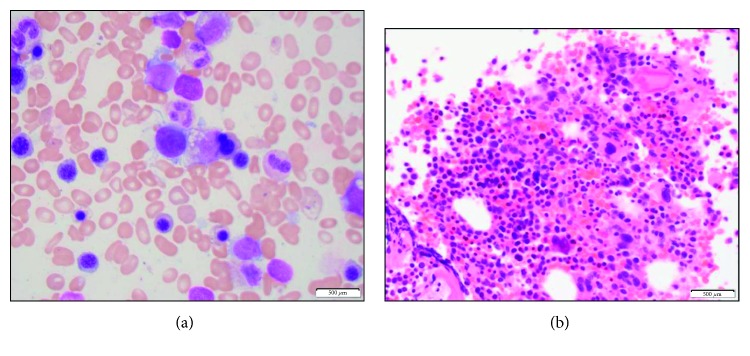
A bone marrow aspirate (a) and core biopsy (b) showing a hypercellular marrow with trilineage dyspoiesis and associated erythroid hyperplasia without any fibrosis.

Flow cytometry showed no immunophenotypic evidence of monoclonal B or atypical T cells and no increase in blasts. Peripheral blood flow cytometry for paroxysmal nocturnal hemoglobinuria clone was negative. A bone marrow aspirate and core biopsy in Figure 2 show a hypercellular marrow with trilineage dyspoiesis and associated erythroid hyperplasia without any fibrosis. A few ringed sideroblasts are identified, comprising 20–25% of erythroid precursors.

**Figure 3:**
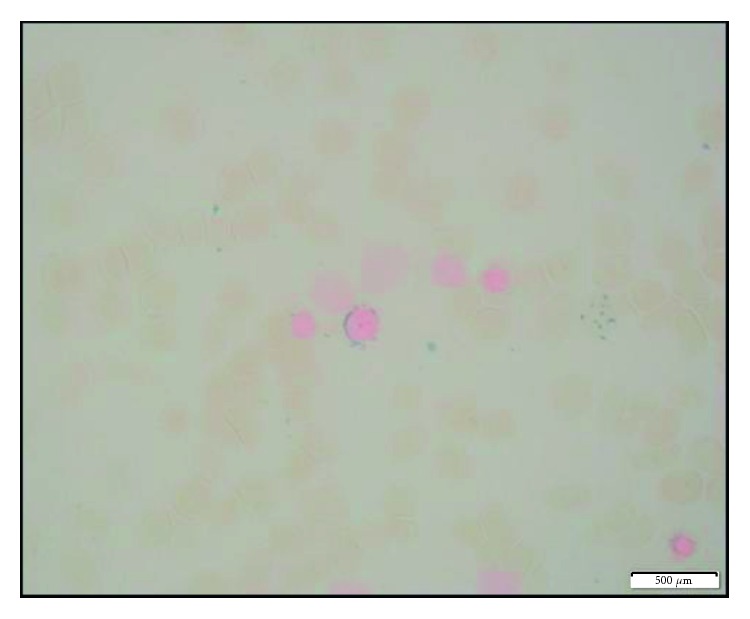
Iron staining before treatment showing increased storage and sideroblastic iron with few ring sideroblastic iron.

Iron staining in Figure 3 shows increased storage and sideroblastic iron with few ring sideroblastic iron, and overall, the findings are most consistent with myelodysplastic syndrome favoring refractory cytopenia with multilineage dysplasia.

**Figure 4:**
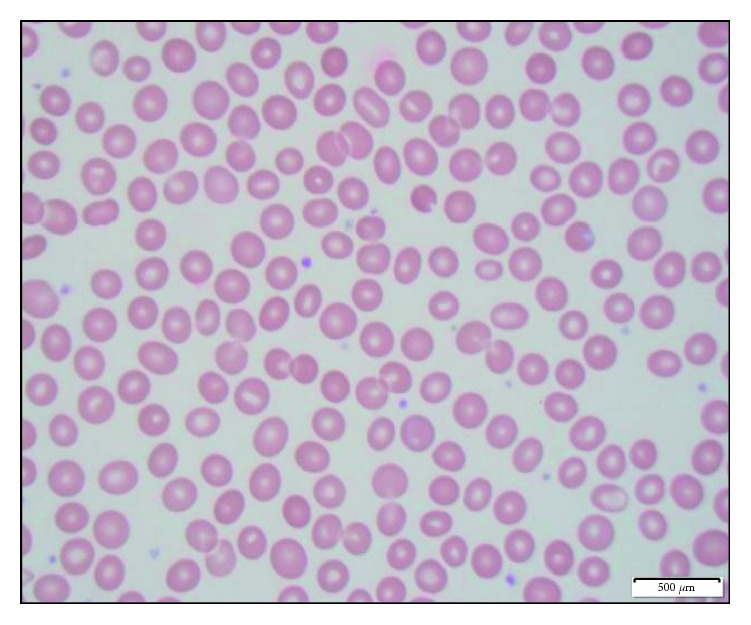
Peripheral smear showing improvement in elliptocytosis after completion of 6 cycles of treatment with azacytidine.

Cytogenetics showed 46,XY,del(20)(q11.2q13.3)[19]/46,XY. His IPSS-R score was 3.5, stratifying him to the intermediate risk group, with an estimated median survival of 3 years. Patient was started on therapy, and he has completed eight cycles of azacytidine, a demethylating agent which helps in hypomethylation of DNA and restoring normal gene differentiation and proliferation. Patient has been independent of any supportive transfusions, and repeat bone marrow biopsy after completion of 6 cycles showed complete cytogenetic remission as well as significant improvement in elliptocytosis as shown in Figure 4. Our patient expired due to recurrent metastatic prostate cancer, but till the end he was responding well to treatment of MDS with azacytidine.

## 3. Discussion

Myelodysplastic syndrome (MDS) comprises a heterogeneous group of malignant stem cell disorders characterized by ineffective blood cell production and an increased risk of transformation to acute leukemia. The disease entity often produces many misshapen red blood cells from the bone marrow characterized by increased number of blasts, dysplastic cells, and ring sideroblast. Elliptocytosis is commonly seen as a hereditary condition. Mutations in protein 4.1, alpha spectrin, beta spectrin, glycophorin C, and band 3 have been reported in hereditary elliptocytosis. While the causal factors leading to acquisition of elliptocytosis in cases of MDS have not been definitively identified, acquired elliptocytosis may be seen in a variety of bone marrow disorders, including architectural disturbances such as fibrosis and myelophthisis. Acquired elliptocytosis is generally characterized by fewer elliptocytes (<15% of total red blood cells) than the marked numbers seen in hereditary elliptocytosis (HE). While elliptocytosis in the setting of MDS has been reported, it is rare for it to be present in the q20 deletion variant.

To our knowledge, rare cases of MDS with the del(20q) variant and elliptocytosis were reported in the literature. The case reported in this paper is now the 16th documented case of acquired elliptocytosis in MDS. Of the 16 cases of MDS with an acquired elliptocytosis, 15 reported cytogenetics, and of those 15 patients, 12 had deletions of chromosome 20q. Although cases have been described in the literature, no studies regarding the course and prognosis of this special subset have been carried out.

Knight and Czuchlewski [1] described a case with acquisition of elliptocytosis as MDS progressed. Kjelland et al. [2] reported a unique case of acquired elliptocytosis presumably related to MDS, with a high percentage of ring sideroblasts and multilineage dysplasia. MDS with ring sideroblasts is a relatively common subtype of MDS, found in approximately 25% of all MDS cases [3]. In our patient also iron staining before treatment showed increased storage with few ring sideroblasts.

Baweja et al. [4] published 2 cases of patients with marked elliptocytosis and MDS with del 20q abnormality reported in the USA. They think elliptocytosis was most likely derived from acquired clones with abnormal development of the erythroid lineage. These patients had indolent course with prolonged history of mild to moderate blood cytopenias and low risk to transformation to acute leukemia. Evaluation for protein 4.1 deficiency in one of these 2 patients was done, but it was pending by the time report was published. Ishida et al. [5] published a similar case, and they analyzed red blood cell membrane proteins by sodium dodecyl sulphate-polyacrylamide gel electrophoresis that showed normal electrophoretic patterns with no quantitative abnormalities of each protein. However, diminished production of protein 4.1 (EBP41), a structural RBC protein implicated in some cases of HE, has also been demonstrated in MDS with elliptocytosis. Hur et al. [6] published a case in which acquired elliptocytosis occurred as an unusual morphological feature of MDS, associated with reduced quantity of protein 4.1 (30% of control) and deletion of chromosome 20q. Unfortunately, testing for protein 4.1 was not approved by insurance in our patient.

Perna et al. [7] reported from animal studies that haploinsufficiency of *L3MBTL1* contributes to some (20q−) myeloproliferative neoplasms and myelodysplastic syndromes by affecting erythroid differentiation. Our case report raises interesting questions: Does MDS with rarely reported elliptocytosis indicate a disease process that is different from the usual 20q deletion? Is haploinsufficiency of *L3MBTL1* responsible for this manifestation? To evaluate this idea, future research should aim at clinical significance of this subtype of MDS with this unusual RBC morphology.

## Figures and Tables

**Table 1:** Patient's characteristics.

Age/sex	73/male
Year of diagnosis of MDS	2015
WBC	1.8 K/cmm
Platelets	36 K/cmm
Hemoglobin	9.3 gm/dl
Hematocrit	28%
Reticulocyte	6.1%
LDH	196 IU/L
Direct Coombs	Negative
BM cytogenetics	46,XY,del(20)(q11.2q13.3) [19]/46,XY
Prior therapy	Tomo radiation of prostate

## References

[1] Knight J., Czuchlewski D. R. (2013). Acquired elliptocytosis of myelodysplastic syndrome. *Blood*.

[2] Kjelland J. D., Dwyre D. M., Jonas B. A. (2017). Acquired elliptocytosis as a manifestation of myelodysplastic syndrome with ring sideroblasts and multilineage dysplasia. *Case Reports in Hematology*.

[3] Navarro I., Ruiz M. A., Cabello A. (2006). Classification and scoring systems in myelodysplastic syndromes: a retrospective analysis of 311 patients. *Leukemia Research*.

[4] Baweja M., Moreno-Aspitia A., Menke D. M., Roy V., Zubair A. (2005). Marked elliptocytosis in myelodysplastic syndromes (MDS) is associated to deletion of chromosome 20q. *Blood*.

[5] Ishida F., Shimodaira S., Kobayashi H. (1999). Elliptocytosis in myelodysplastic syndrome associated with translocation (1;5)(p10;q10) and deletion of 20q. *Cancer Genetics and Cytogenetics*.

[6] Hur M., Lee K. M., Cho H. C. (2004). Protein 4.1 deficiency and deletion of chromosome 20q are associated with acquired elliptocytosis in myelodysplastic syndrome. *Clinical and Laboratory Haematology*.

[7] Perna F., Gurvich N., Hoya-Arias R. (2010). Depletion of *L3MBTL1* promotes the erythroid differentiation of human hematopoietic progenitor cells: possible role in 20q− polycythemia vera. *Blood*.

